# Spectrometry and Its Application for the Detection of RNA‐Binding Proteins: Advancements, Techniques and Challenges

**DOI:** 10.1002/ansa.70026

**Published:** 2025-08-06

**Authors:** Mina Moradi, Zahra Farjami, Mohammad Mehdi Akbarin

**Affiliations:** ^1^ Department of Chemistry, Faculty of Sciences Ferdowsi University of Mashhad Mashhad Iran; ^2^ Department of Modern Sciences and Technologies, Faculty of Medicine Mashhad University of Medical Sciences Mashhad Iran; ^3^ Inflammation and Inflammatory Diseases Research Center Medical School Mashhad University of Medical Sciences Mashhad Iran; ^4^ Medical School, Medical Sciences Islamic Azad University Mashhad Iran

**Keywords:** long non‐coding RNA (lncRNA), matrix‐assisted laser desorption/ionization time‐of‐flight (MALDI‐TOF), mass spectrometry, pulldown assay, RNA‐binding protein, spectrometry

## Abstract

Spectrometry is a fascinating field of analytical science that encompasses a range of techniques used to study the interaction between electromagnetic radiation and matter. Through the measurement and analysis of various radiations, spectrometry provides valuable insights into the composition, structure and properties of different substances. Spectrometry allows for the identification and quantification of proteins based on their characteristics and abundance. By comparing the mass spectrometry data obtained from the pulldown assay with databases of known proteins, it is possible to identify the interacting proteins with high confidence. Long non‐coding RNAs (lncRNAs), as one of the most important RNA‐binding proteins, have emerged as key players in gene regulation, with nearly 80% of transcripts in human cells being lncRNA species. These nonprotein‐coding transcripts, longer than 200 nucleotides, have shown great potential in various biological processes and diseases. However, their functional characterization remains a challenge due to their lower expression levels and the limitations of current techniques. Therefore, in this study, we aim to review spectrometry and its diverse types for application in the determination of general properties of RNA‐binding proteins.

## 1 Introduction

### 1.1 Spectrometry: Analysing the Characteristics of Light

Spectrometry is the measurement and analysis of the different characteristics of light. Spectrometry is a scientific method used to measure the interaction between matter and electromagnetic radiation [1, 2]. Atoms and molecules absorb light at different frequencies, and these absorption lines can be used to uniquely identify them.

### 1.2 Basic Principles

**FIGURE 1:**
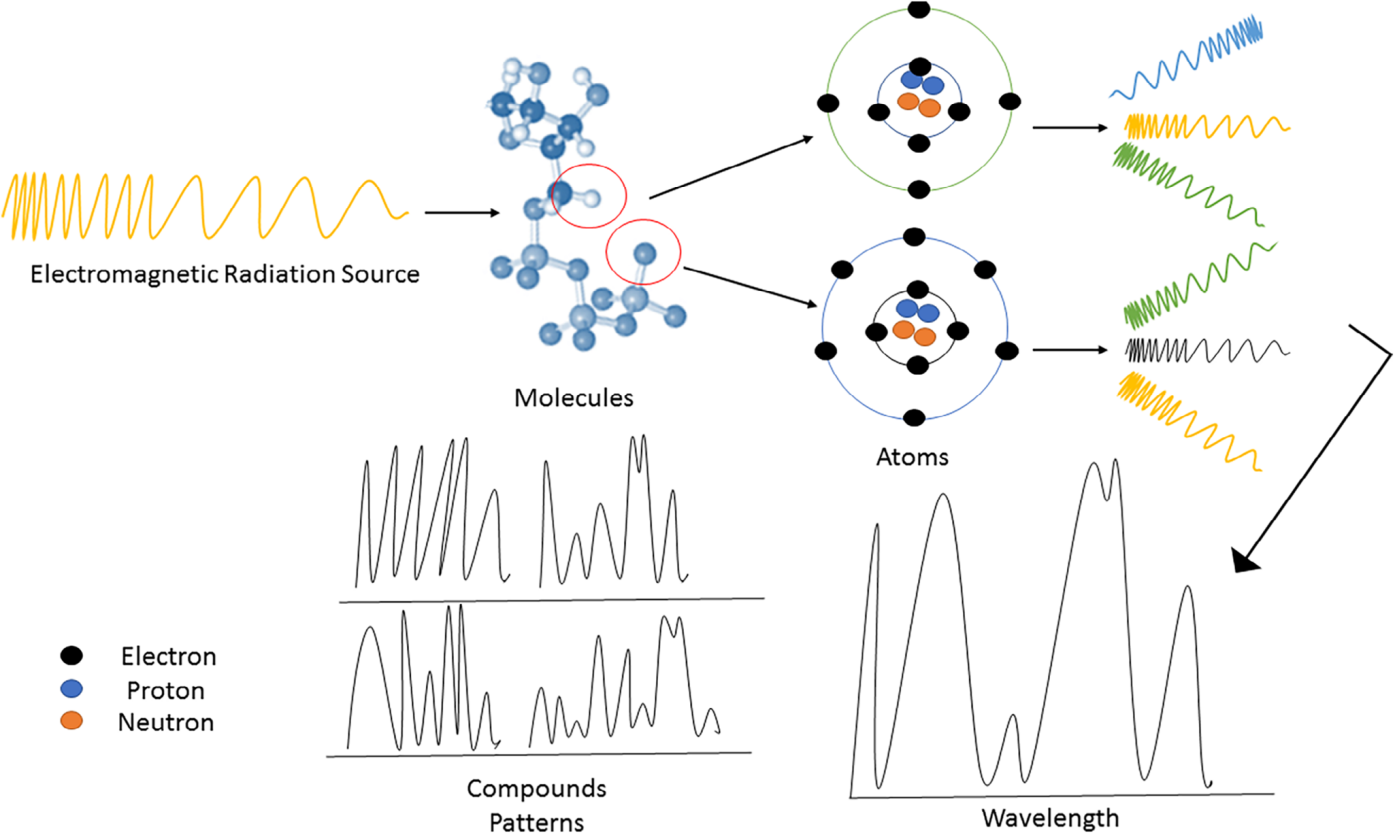
The basic principle of spectrometry for detection and identification of material compounds based on absorbing, reflecting or transmitting the different wavelengths of electromagnetic radiations.

Spectrometry is based on the fundamental principle that materials interact with electromagnetic radiation, such as light, and absorb, reflect or transmit the different wavelengths of light by the atoms or molecules that make up the material [2]. By analysing the way in which the radiation is absorbed or reflected, it is possible to determine the chemical composition and structural properties of the material (Figure 1). Therefore, spectrometry can be used to identify and quantify the chemical composition of a sample, as well as to determine its physical properties [2].

### 1.3 Applications

Spectrometry is a powerful analytical technique widely used across various scientific fields, including astronomy, chemistry, biology and environmental science. Fluorescence spectroscopy can be used to determine the conformational changes of proteins, whereas circular dichroism spectroscopy is used to analyse the secondary structure of proteins [3, 4, 5].

Spectrometry is also used in environmental science for monitoring air and water quality [6, 7]. For example, Fourier transform infrared (FTIR) spectroscopy is used to analyse pollutants in the air [8], whereas ultraviolet (UV)–visible spectroscopy is used to detect pollutants in water [9, 10].

It allows scientists to analyse the different characteristics of light and obtain valuable information about the chemical composition and physical properties of different substances and structures. Advancements in technology have made it possible to measure and analyse increasingly complex samples, making spectrometry an even more valuable tool for scientific research.

### 1.4 Mass Spectrometry (MS): Basic Principles and Applications

MS is an analytical method used to determine the molecular mass and composition of chemical compounds. It works by ionizing molecules and then separating them according to their mass‐to‐charge ratio (*m*/*z*) using an electric and/or magnetic field [11]. MS has a wide range of applications in areas, such as protein sequencing, drug discovery, environmental monitoring and forensic science, among others [11, 12].

#### 1.4.1 Basic Principles

The basic elements of an MS are an ionization source, an analyser and a detector. The sample is first ionized by the ionization source, which can be a laser, electron beam or chemical reagent. This produces positively or negatively charged ions, which are then introduced into the analyser, where they are separated according to their *m*/*z* ratio [11, 13].

Magnetic sector, quadrupole, time‐of‐flight (TOF) and ion trap analysers are all commonly used in MS. Each analyser operates on different principles, allowing it to be used for specific applications. For example, TOF analysers are often used for large molecules, whereas ion trap analysers are better suited for small molecules.

After separation, the ions are detected by the detector. This produces a mass spectrum, which is a plot of the relative abundance of ions versus their *m*/*z* ratio.

#### 1.4.2 Applications

MS has a wide range of applications because it can be used to analyse a large variety of molecules, including small molecules, peptides, proteins and nucleic acids [12, 14, 15, 16]. One of the most common applications of MS is in proteomics, where it is used to identify proteins by analysing their peptide fragments [13]. MS is also an important tool in drug discovery, where it is used to identify potential drug candidates as well as to determine the purity of drug compounds.

MS is also widely used in the environmental sciences to monitor pollutants and identify unknown compounds in the environment. It is particularly useful for analysing volatile organic compounds (VOCs) and semi‐VOCs (SVOCs) in air pollution and water contamination studies [17]. Therefore, MS's ability to characterize complex mixtures and identify unknown compounds has made it an indispensable tool in drug discovery, proteomics, environmental monitoring and forensic science. Continuous developments in MS technology have made it possible to analyse increasingly complex samples and identify even smaller quantities of compounds, making it an even more valuable tool for scientific research.

## 2 Magnetic Sector MSs

Magnetic sector MSs, also known as magnetic sector instruments, are MSs used in analytical chemistry. They consist of an ion source, a mass analyser and an ion detector [18]. The ion source produces gaseous ions, which are then separated according to their mass‐to‐charge ratio in the mass analyser. The ion detector records the ions as they emerge from the mass analyser [18, 19].

## 3 Working Principles

**FIGURE 2:**
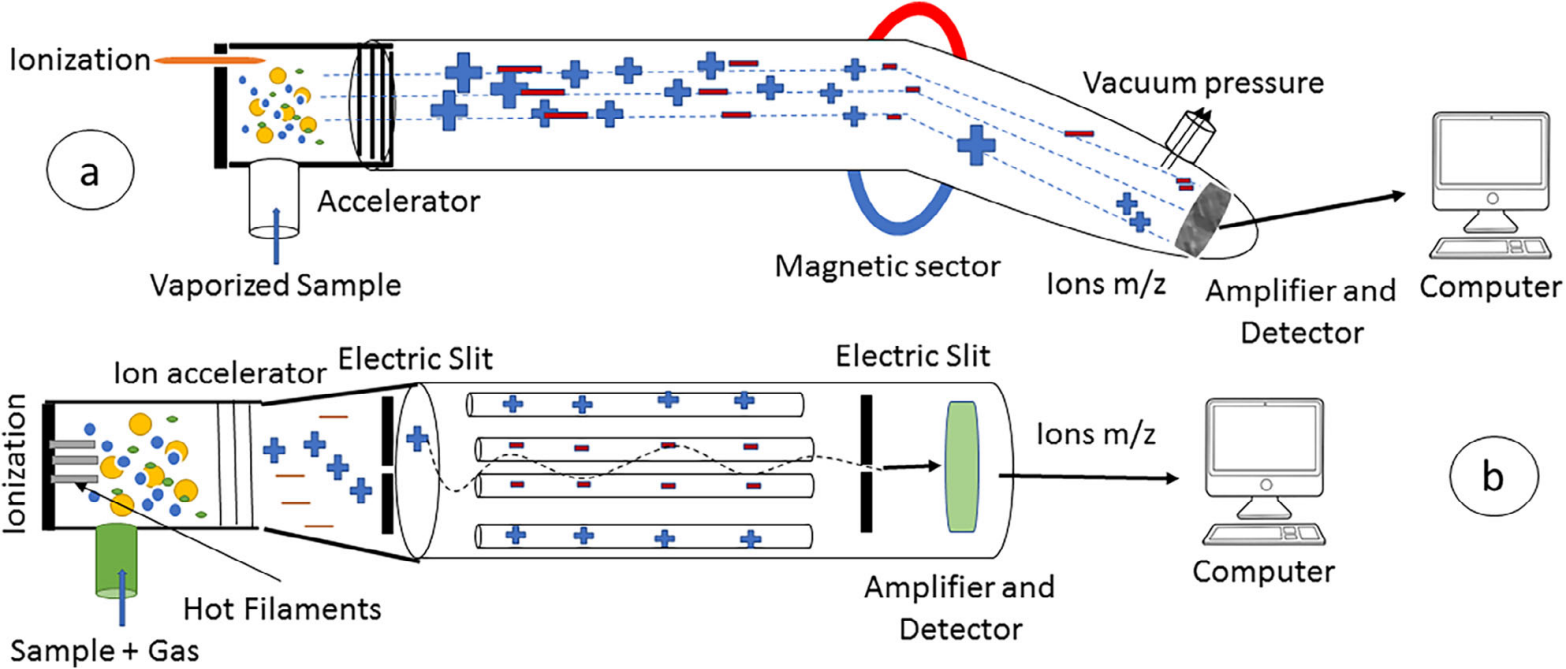
The schematic illustration of mass spectrometry by the magnetic sector (a) and quadrupole (b).

Magnetic sector MSs work by using the principles of magnetic deflection and electric deflection to separate ions based on their mass‐to‐charge ratio (Figure 2a). The ions are first accelerated by an electric field and then deflected by a magnetic field. The deflection angle of the ions depends on their mass‐to‐charge ratio. The ions are then detected, and counted by a detector [18, 20].

Magnetic sector MSs are used in a wide range of applications in analytical chemistry, including the identification of unknown compounds, the determination of analyte concentrations in samples and the characterization of biomolecules [18, 19, 20]. They are also used in environmental analysis, drug metabolism studies and forensic science.

### 3.1 Quadrupole MS

Quadrupole MS is a type of mass analyser used in MSs (Figure 2b). It consists of four parallel rods, called quadrupole rods, mounted on a central axis. An electric field is applied to the rods, causing the ions in the gas phase to travel through the quadrupole [21]. The strength of the electric field is adjusted so that the ions are focused into a narrow beam. The ions are then detected and the intensity of each ion is analysed to determine the *m*/*z* ratio [21].

## 4 Applications

Quadrupole MS is used in a wide range of applications, including proteomics, metabolomics, environmental analysis and drug discovery. In proteomics, quadrupole MS is used to separate and identify proteins [22, 23]. With its high sensitivity, high resolution and high speed, it is a powerful tool for analysing chemical species.

### 4.1 Ion Trap Spectrometry

Ion trap spectrometry is an analytical technique that uses ions trapped in an electromagnetic field to analyse samples. It involves trapping ions in an electric field, then measuring their mass‐to‐charge ratio and finally analysing their chemical properties [24, 25].

Ion trap spectrometry is a versatile technique and can be used to analyse a wide range of samples, including biological samples, environmental samples and forensic samples [25, 26]. It is often used for determining the identity of unknown compounds, as well as for trace analysis.

Ion trap spectrometry has several advantages over other types of spectrometry, such as gas or liquid chromatography. It is fast, simple and relatively inexpensive and often used in conjunction with other analytical techniques to provide a more complete picture of a sample's chemical makeup [24, 27, 28]. It is a valuable tool for chemists and scientists in many industries, including pharmaceuticals, chemical manufacturing and food processing.

### 4.2 Time‐of‐Flight

TOF MS is a powerful analytical technique used to determine the mass‐to‐charge ratio of ions [29]. It works by measuring the time it takes for ions to travel a certain distance in an electric field. The ions are first ionized and then accelerated into a flight tube, where they are separated on the basis of their mass‐to‐charge ratio. The ions are then detected at the end of the flight tube, and the resulting data can be used to identify the chemical composition of the sample being analysed [29].

TOF MS is particularly useful for analysing large molecules, such as proteins and peptides, because it can accurately measure their mass‐to‐charge ratio. It is also a very sensitive technique, capable of detecting very low concentrations of analytes [29, 30]. TOF MS is used in a wide range of applications, including proteomics, metabolomics and drug discovery.

### 4.3 Matrix‐Assisted Laser Desorption/Ionization TOF (MALDI‐TOF) MS

MALDI‐TOF MS is an analytical technique that revolutionizes the identification and analysis of biomolecules, such as peptides, lipids, saccharides and other organic macromolecules [31]. This powerful technology overcomes the limitations of traditional identification methods, providing accurate and rapid results in various fields, including proteomics, metabolomics, lipidomics and glycomics [32, 33, 34]. By ionizing samples into charged molecules and measuring their mass‐to‐charge ratio (*m*/*z*), MALDI‐TOF MS enables the determination of molecular weight, identification of protein sequences, recognition of protein structures, quantification of protein content and analysis of glycan structures [33, 35, 36].

### 4.4 The Principle of MALDI

**FIGURE 3:**
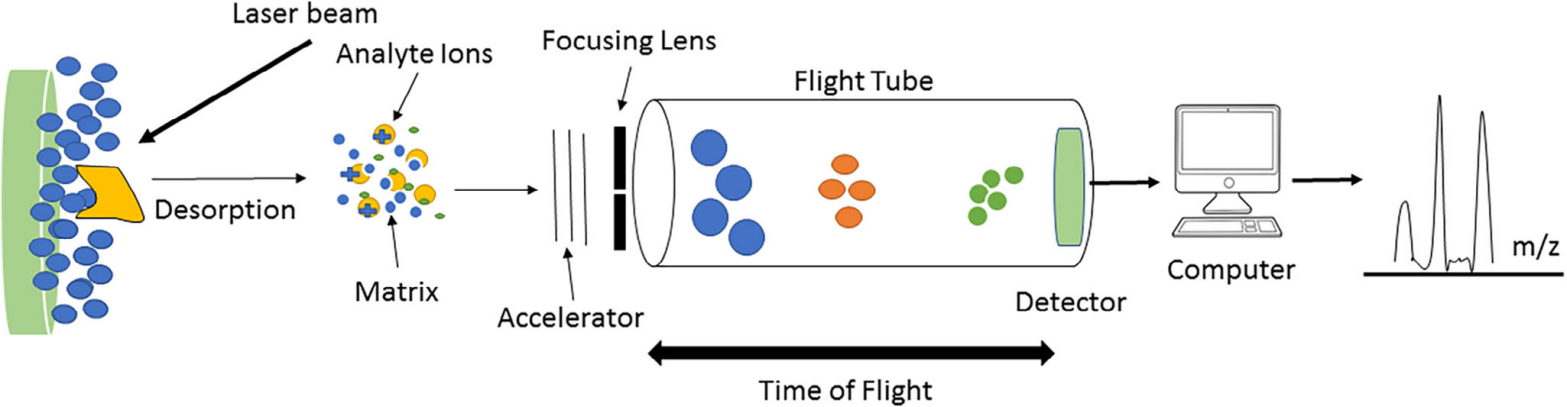
The basic principle of MALDI‐TOF mass spectrometry. The sample will be dried in desired matrix and introduced in MALDI plate. Then the laser beam will be ionized and desorbed analyte and matrix molecules. These molecules will be accelerated and separated in flight tube based on *m*/*z* index. The component material of sample will be uncertificated by their *m*/*z* index and different in time‐of‐flight.

The principle of MALDI involves the use of a MALDI technique, which allows the ionization of large molecules without significant fragmentation [31]. In this process, a sample is mixed with a suitable matrix material and applied to a metal plate. A pulsed laser irradiates the sample, triggering ablation and desorption of the sample and matrix material. The analyte molecules are then ionized by being protonated or deprotonated in the hot plume of ablated gases. This ionization process typically results in the analyte molecules carrying a single positive charge (Figure 3). The resulting ions can be accelerated into a mass analyser for further analysis [31].

### 4.5 Types of Laser Used in MALDI

MALDI utilizes lasers of both UV and IR wavelengths [37, 38]. However, UV lasers are the most commonly used light sources in analytical MALDI. These UV lasers provide the necessary energy to ionize the analyte molecules while minimizing fragmentation [38, 39]. The absorption of laser energy by the matrix material facilitates the ionization of large molecules, ensuring efficient analysis without compromising the integrity of the sample.

## 5 Benefits of MALDI‐TOF MS

### 5.1 Rapid and Accurate Identification of Microorganisms

In the clinical laboratory, MALDI‐TOF MS is replacing traditional methods for identifying microorganisms [40]. This technique overcomes the challenges associated with time‐consuming and complex identification processes. By comparing the spectra of unknown organisms with a database of known organisms, MALDI‐TOF MS enables the rapid and accurate identification of bacteria and fungi [40, 41]. The expansion of databases containing spectra of known organisms has enhanced the identification of species with similar phenotypic, genotypic and biochemical properties. This advancement in clinical care has improved the diagnosis of infections caused by relatively rare species, decreased the time to diagnosis and resulted in a reduction in the time to appropriate therapy and hospital stays.

### 5.2 Cost‐Effective and Time‐Efficient Analysis

One of the significant advantages of MALDI‐TOF MS is its cost‐effectiveness and time efficiency. Traditional methods for identification and analysis often require time‐consuming and complex procedures, such as subculturing and subjective interpretation of phenotypic characteristics. In contrast, MALDI‐TOF MS provides rapid results, eliminating the need for multiple subcultures and reducing the turnaround time for identification [42, 43]. This efficiency translates into cost savings and improved patient care, as timely and accurate diagnoses enable the prompt administration of appropriate therapy.

### 5.3 Versatility in Omics Fields

MALDI‐TOF MS finds versatile applications in various omics fields, including proteomics, metabolomics, lipidomics and glycomics. In proteomics, this technique allows the analysis of complex protein mixtures and facilitates the determination of protein molecular weight, identification of protein sequences, recognition of protein structures and quantification of protein content [44, 45].

In metabolomics, MALDI‐TOF MS enables the identification and quantification of metabolites, including lipid‐related molecules. By analysing the lipid composition, lipid metabolites and other lipid‐related molecules, this technique provides valuable insights into lipidomics research [46, 47, 48]. Additionally, MALDI‐TOF MS plays a crucial role in glycomics, allowing the study of glycans and their structures. It aids in the analysis of glycosylation and glycan structure, contributing to advancements in glycomics research [49, 50].

### 5.4 Long Non‐Coding RNAs (lncRNA) and Its Biological Functions

lncRNAs are non‐coding transcripts, which are longer than 200 nucleotides, and were initially discovered over two decades ago through genetic studies. Since then, the advent of high‐throughput sequencing technologies has further shed light on the importance of lncRNAs in diverse biological contexts [51, 52]. Recent studies have revealed that lncRNA species make up nearly 80% of all transcripts in human cells. These transcripts can be categorized on the basis of their genomic location, including large intergenic non‐coding RNAs, natural antisense transcripts, pseudogenes, long intronic ncRNAs and other divergent transcripts [51, 52]. lncRNAs regulate gene expression through four primary functional archetypes: signals, decoys, guides and scaffolds, each representing a unique mechanism of molecular interaction [53, 54]. As signals, lncRNAs like *COLDAIR* and *COOLAIR* are transcribed in response to environmental cues, such as cold, and initiate processes like flowering through epigenetic regulation involving PRC2 [55, 56]. As decoys, lncRNAs such as *Gas5* sequester regulatory proteins like the glucocorticoid receptor, thereby preventing gene activation [57]. In their guide role, lncRNAs like *HOTTIP* recruit chromatin‐modifying complexes (e.g., MLL‐1) to specific genomic loci, influencing gene expression patterns during development [58, 59]. As scaffolds, lncRNAs such as *HOTAIR* bring together complexes like PRC2 and LSD1 to coordinate chromatin modifications [58, 59]. These archetypes likely evolved in a step‐wise manner—beginning with simple transcriptional signals and gradually acquiring binding capacity and structural complexity—enabling lncRNAs to mediate intricate regulatory networks and contribute to biological diversity [54].

### 5.5 Complexity and Evolution of lncRNA Function

The simple signal archetype, represented by lncRNAs such as enhancer RNAs (eRNAs), requires the transcription of regulatory DNA elements [54]. As lncRNAs gain the ability to bind proteins through RNA motifs, they transition into molecular decoys. Subsequent acquisition of target specificity allows lncRNAs to become guides, directing effectors to specific DNA sequences. With the duplication, fusion and recombination of nucleic acids, lncRNAs may evolve into scaffolds, capable of assembling multiple molecular complexes [54].

This step‐wise scenario is supported by experimental evidence, which demonstrates the feasibility of evolving new lncRNA regulators. RNA evolution, with its diverse structural and functional possibilities, provides a means for lncRNAs to mediate processes involving proteins with cationic patches. The evolutionary plasticity of lncRNAs suggests that they may play a significant role in the fine‐tuning of gene expression and the development of complex biological systems.

### 5.6 Implications for Disease and Future Perspectives

The study of lncRNAs has significant implications for understanding the molecular basis of human diseases. Increasing evidence suggests that polymorphisms and mutations in lncRNA regulatory regions are associated with various disorders [51, 60]. As research progresses, uncovering the altered fine‐tuning mechanisms in different anomalies may provide valuable insights for disease management and treatment.

Exploring the mechanisms through which physiological and environmental changes are translated into altered gene function through lncRNAs and their regulatory networks is an area ripe for future exploration. As more examples of lncRNA‐mediated gene regulation are discovered, the versatility of these large transcripts as genetic regulators may rival that of small RNAs and proteins. The archetypal classifications of lncRNAs discussed in this article provide a useful framework for understanding their diverse functions and evolutionary origins.

As research progresses, further investigation into the molecular mechanisms and evolutionary origins of lncRNAs will deepen our understanding of gene regulation and its implications for human health and disease.

Although it is believed that many lncRNAs have functional roles, only a small proportion has been proven to be biologically and physiologically relevant. This is due to their lower expression levels and limitations in current techniques for studying lncRNAs. To better understand the functions of lncRNAs, new assays and methods are being developed, such as the lncRNA pulldown assay, which has been used to identify their interacting protein partners in cellular contexts [61].

### 5.7 Genome‐Wide Approaches for Studying lncRNAs

Several genome‐wide approaches have been developed to examine the genome localization of lncRNAs and their interactions with proteins. Two commonly used methods are genome‐wide chromatin immunoprecipitation‐sequencing (ChIP‐seq) and RNA immunoprecipitation followed by sequencing (RIP‐seq) [62, 63]. ChIP‐seq allows researchers to map the binding sites of lncRNAs and their interacting proteins on the genome. This method provides insights into the regulatory roles of lncRNAs in gene expression. On the other hand, RIP‐seq enables the identification of lncRNAs that interact with specific proteins, shedding light on their potential functions [64].

### 5.8 Application of MS in Detection of lncRNA

One of the key advantages of MS for studying lncRNAs is its ability to detect and analyse post‐translational modifications (PTMs) of these molecules [65]. PTMs are modifications that occur after a protein or RNA has been synthesized and can include phosphorylation, methylation and acetylation [66, 67]. These modifications can have a profound impact on the function and activity of lncRNAs, and MS can be used to identify and quantify these modifications.

Another advantage of MS for studying lncRNAs is its ability to analyse the interactions between lncRNAs and other molecules, such as proteins, DNA and RNA. These interactions are critical for the function and activity of lncRNAs, and MS can be used to identify and quantify the proteins and other molecules that interact with lncRNAs.

In recent years, there has been growing interest in understanding the role of lncRNAs in cancer progression. Researchers are constantly exploring new avenues to understand the underlying mechanisms driving cancer progression. In recent years, lncRNAs have emerged as key players in various aspects of cancer biology [68]. These non‐coding RNA molecules have been implicated in tumour development, metastasis and response to therapy [69, 70].

### 5.9 The lncRNA Pulldown Assay

**FIGURE 4:**
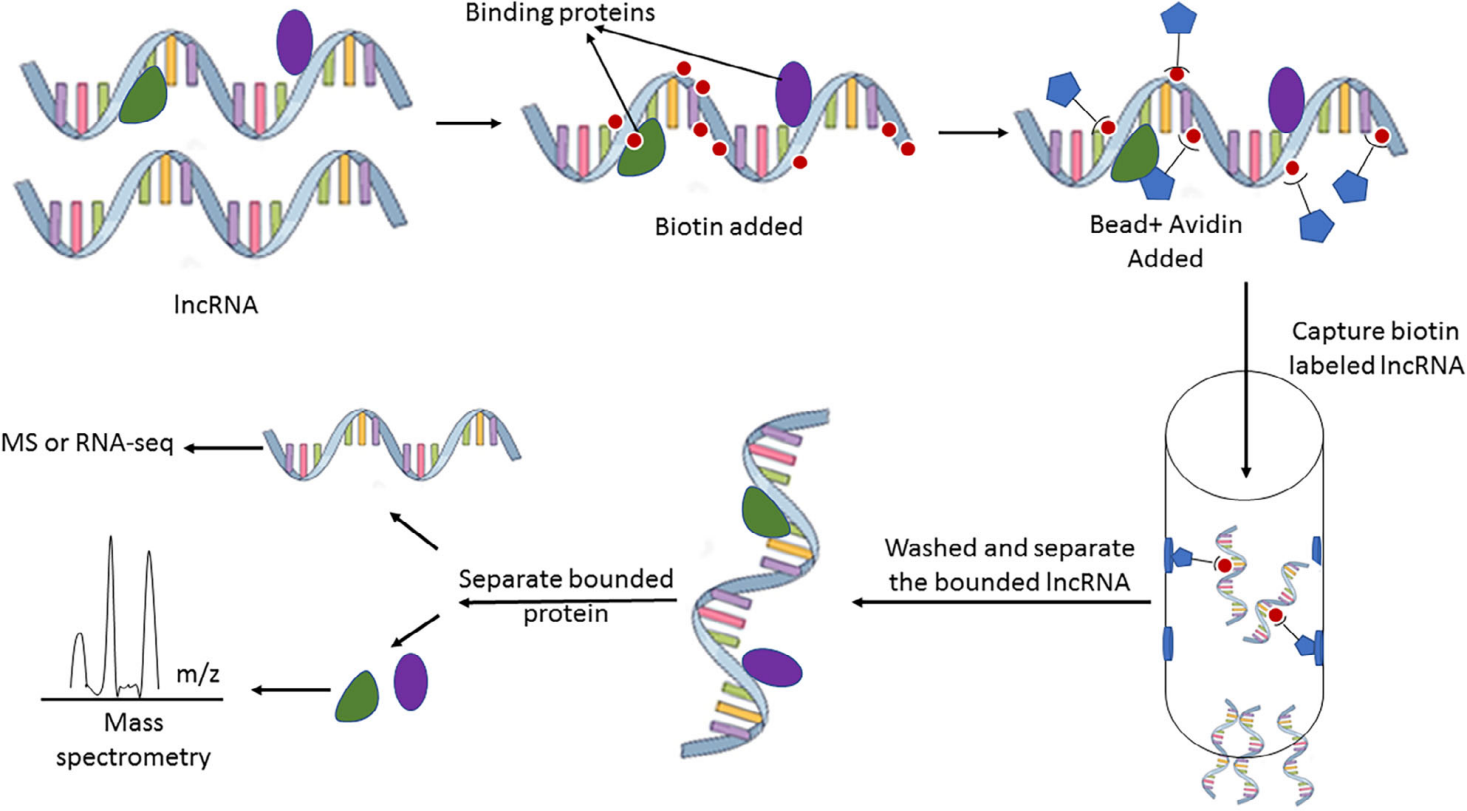
The schematic demonstration of lncRNA pulldown assay technique. In this method, the RNA and its bound proteins will be biotin–avidin labelled and separated with a magnetic bead. Then bound RNA will be washed, and its related protein will be identified by mass spectrometry methods such as MALDI‐TOF. lncRNA, long non‐coding RNA; MS, mass spectrometry.

The lncRNA pulldown assay is an open‐ended method that has been frequently used to identify the interacting protein partners of lncRNAs in cellular contexts [61]. This assay involves the isolation of lncRNAs and their associated protein complexes using biotinylated antisense oligonucleotides complementary to the lncRNA of interest (Figure 4). The biotinylated antisense oligonucleotides are then immobilized on streptavidin‐coated magnetic beads, allowing for the pulldown of the lncRNA–protein complexes [61]. The interacting proteins are subsequently identified using MS, a technique that analyses the mass and charge of molecules to determine their identities. In the follow‐up, we reviewed some of these studies, particularly around cancer genomics, where the role of lncRNA and its interaction with binding proteins are demonstrated by pulldown assay.

### 5.10 CALA: Unravelling the Interactions and Subcellular Localization

One of the lncRNAs that has garnered attention in recent studies is *CALA* [71]. Research has revealed that *CALA* interacts with multiple ribonucleoproteins (RNPs) and primarily associates with cytoplasmic G3BP1 [71]. Ribosome foot printing and expression data analysis of the *CALA* gene locus have provided insights into its potential involvement in cellular processes. Additionally, sucrose density gradient ultracentrifugation and antisense affinity selections have demonstrated the recovery and enrichment of *CALA* from cell lysates.

The interaction between *CALA* and G3BP1 has been validated through anti‐G3BP1 RNA immunoprecipitations (RIPs). These experiments have provided evidence of the CALA–G3BP1 interaction in whole cell lysates [71]. Moreover, subcellular localization studies have highlighted the presence of *CALA* in the cytoplasm, further emphasizing its potential role in cytoplasmic processes. The comprehensive characterization of *CALA's* interactions and subcellular localization provides a foundation for understanding its functional relevance in cancer biology.

### 5.11 ALT1: Unveiling a Novel Regulator of Vascular Endothelial Cell Cycle

Vascular endothelial cells play crucial roles in angiogenesis and vascular homeostasis. The lncRNA *ALT1* has recently been identified as a novel regulator of the cell cycle in human umbilical vein endothelial cells (HUVECs) [72]. *ALT1* expression has been found to be downregulated in growth‐arrested and contact‐inhibited HUVECs.

Functional studies have demonstrated that knocking down *ALT1* leads to G0/G1 cell cycle arrest in HUVECs, indicating its role in promoting proliferation. Further investigations have identified angiotensin‐converting enzyme 2 (ACE2) as a direct target gene of *ALT1* [72]. Knockdown of *ALT1* or ACE2 results in decreased expression of cyclin D1, a key regulator of the cell cycle. The enhanced ubiquitination and degradation of cyclin D1, possibly involving HIF‐1α and protein von Hippel–Lindau (pVHL), have been implicated in this process [72].

### 5.12 Colon Cancer–Associated Transcript 1 (CCAT1): Unravelling the Protein Interactions Underlying Tumour Metastasis

*CCAT1* has been implicated in the progression of various cancers [73]. Although previous studies have primarily focused on the decoy function of *CCAT1* for microRNAs (miRNAs), the regulatory mechanisms of *CCAT1*–protein interactions in tumour metastasis remain largely unknown [73].

Proteome‐wide analysis using RNA antisense purification coupled with MS has revealed numerous proteins that interact with *CCAT1*. Among these proteins, vimentin has been identified as a significant binding partner of *CCAT1* [73]. Functional studies have demonstrated that *CCAT1* stabilizes vimentin protein, thereby enhancing cancer cell migration and invasion abilities.

### 5.13 Gallbladder Cancer and Autophagy Regulation

Gallbladder cancer is a common malignancy of the biliary tract and is known for its resistance to chemotherapy. In a study by Cai, et al., the researchers investigated the role of lncRNA in autophagy and chemoresistance of gallbladder cancer cells [74]. They discovered an lncRNA called gallbladder cancer drug resistance–associated lncRNA1 (*GBCDRlnc1*), which was found to be upregulated in gallbladder cancer tissues [74]. Through knockdown experiments, the researchers demonstrated that inhibiting *GBCDRlnc1* enhanced the sensitivity of Dox‐resistant gallbladder cancer cells to chemotherapy. Further mechanistic studies revealed that *GBCDRlnc1* interacts with phosphoglycerate kinase 1, leading to the downregulation of the autophagy initiator ATG5–ATG12 conjugate [74]. These findings suggest that *GBCDRlnc1* could be a potential therapeutic target for the treatment of advanced gallbladder cancer.

### 5.14 Melanoma and Immune Surveillance

Melanoma, a highly aggressive form of skin cancer, has been the focus of numerous studies investigating the role of lncRNAs in T cell immunosurveillance [75]. In a study by Guillaume L. Gracia‐Maldonado et al., the researchers identified two highly specific melanoma antigens, MELOE‐1 and MELOE‐2, which are produced by the lncRNA and polycistronic RNA, meloe. Interestingly, they also discovered a novel open reading frame (ORF) called MELOE‐3, which is translated exclusively by the classical cap‐dependent pathway [75]. Although MELOE‐1 and MELOE‐2 showed melanoma‐restricted expression and high immunogenicity, MELOE‐3 exhibited poor immunogenicity and was expressed in both melanoma cells and normal melanocytes [75]. These findings suggest that the IRES‐dependent MELOE‐1 and MELOE‐2 could be potential targets for immunotherapy of melanoma.

### 5.15 Hepatocellular Carcinoma (HCC) and TGLC15

HCC is a common malignancy of the liver, particularly prevalent in China [76, 77]. In a study by Chen et al., a novel intergenic lncRNA called *TGLC15* was identified as a potential driver of HCC progression [76]. *TGLC15* was found to be significantly overexpressed in tumour tissues and HCC cell lines. Its higher expression levels correlated with advanced malignant characteristics such as TNM stages, tumour size and metastasis. Functional assays revealed that *TGLC15* promoted HCC migration and viability [76]. In vivo experiments further supported its role in tumour growth and proliferation. Mechanistically, *TGLC15* was found to interact with the transcription factor Sox4, stabilizing it by inhibiting proteasome‐mediated degradation [76]. These findings suggest that *TGLC15* could serve as a potential therapeutic target for HCC intervention. Another lncRNA, *LINC01123*, has been implicated in the progression of HCC [69, 78]. These studies collectively emphasize the significance of lncRNAs in cancer biology and their potential as therapeutic targets.

### 5.16 lncRNAs in Breast Cancer

Breast cancer is a highly prevalent cancer type, and understanding its molecular mechanisms is crucial for improved diagnosis and treatment [79]. The dysregulation of lncRNAs has been observed in breast cancer, but their specific roles in disease progression and metastasis are still not fully understood. A study by Jiang et al. aimed to identify novel breast cancer‐associated lncRNAs using a high‐density SNP array‐based approach. They discovered that high levels of the lncRNA *LincIN* were frequently observed in breast tumours compared to adjacent normal tissues [79]. Importantly, high expression of LincIN was associated with aggressive breast cancer and poor overall survival in patients. Functional experiments demonstrated that *LincIN* knockdown inhibited tumour cell migration and invasion in vitro, suggesting its potential as a therapeutic target for breast cancer [79].

Triple‐negative breast cancer (TNBC) is a subtype of breast cancer that lacks the expression of oestrogen receptor (ER), progesterone receptor (PR) and human epidermal growth factor receptor 2 (HER2) [80]. In a study focused on TNBC by Zhang et al., a novel oncogenic lncRNA called *OLBC15* was discovered. *OLBC15* was found to be highly expressed in TNBC, and its overexpression promoted cell viability and migration in breast cancer cells [80]. In vivo experiments confirmed that *OLBC15* accelerated tumour growth and metastasis. Further investigation revealed that *OLBC15* interacted with the tumour suppressor ZNF326, leading to its destabilization and degradation through enhanced ubiquitination [80]. Notably, clinical analysis showed a negative correlation between *OLBC15* and ZNF326 protein expression in patient samples. These findings suggest that *OLBC15* could be a potential target for therapeutic intervention in TNBC.

### 5.17 lncRNAs in Gastric Cancer

Gastric cancer is a leading cause of cancer‐related deaths worldwide, and the identification of molecular mechanisms involved in its development and progression is essential for improved management [81]. A study by Zheng et al. investigated the mechanism of the lncRNA plasmacytoma variant translocation 1 (*PVT1*) in gastric cancer caused by *Helicobacter pylori* (HP) infection [81]. They found that *PVT1* was significantly upregulated in HP‐infected normal gastric epithelial cells. Knockdown of *PVT1* in gastric cancer cells led to the inhibition of inflammatory cytokines such as tumour necrosis factor‐α (TNF‐α), interleukin (IL)‐1β, IL‐6 and IL‐8. *PVT1* knockdown also inhibited gastric cancer cell migration [81]. The study suggested that *PVT1* may act as a pro‐inflammatory factor and regulate gastric cancer caused by HP infection.

### 5.18 lncRNAs in Nasopharyngeal Carcinoma (NPC)

NPC is a unique malignant cancer with high metastatic potential. The early symptoms of NPC are often not obvious, leading to late‐stage diagnosis and treatment challenges [81]. A study by Chunmei Fan et al. investigated the role of the lncRNA *LOC284454* in NPC. They found that *LOC284454* was upregulated in NPC tissues and associated with poor prognosis. In vitro and in vivo experiments revealed that *LOC284454* promoted the migration and invasion capacity of NPC cells [81]. Further analysis suggested that *LOC284454* modulated the Rho/Rac signalling pathway, which is involved in cytoskeletal dynamics and cell adhesion. This study highlights the potential of *LOC284454* as a novel treatment target and diagnostic/prognostic marker in NPC [81].

To unravel the molecular mechanisms underlying the effects of *LINC01123*, researchers used Coomassie blue staining, RNA pull‐down and MS to identify proteins that interact with *LINC01123*. Their analysis revealed an interaction between *LINC01123* and SRSF7 [82]. This interaction suggests that *LINC01123* may regulate SRSF7 activity, thereby promoting colorectal cancer progression [82]. Further studies are needed to elucidate the precise mechanisms through which *LINC01123* and SRSF7 interact. The MS was done to provide the proteomics data. Their findings revealed that *LINC01123* is upregulated in colorectal cancer tumour tissues, suggesting its potential importance in disease progression. This upregulation could serve as a diagnostic biomarker for colorectal cancer and aid in the development of targeted therapies.

### 5.19 The lncRNA and Its Role in Non‐Small Cell Lung Cancer (NSCLC)

NSCLC is a prevalent and life‐threatening malignancy worldwide. Extensive research has shed light on the crucial role of lncRNAs in NSCLC progression.

By conducting lncRNA profiling, the researchers identified several novel lncRNAs associated with NSCLC. Among these, *LNBC3* exhibited marked overexpression in tumour tissues and NSCLC cell lines [83]. The elevated levels of *LNBC3* were found to correlate with advanced TNM stages, larger tumour size and metastasis. Functional assays demonstrated that *LNBC3* promoted NSCLC migration and viability in vitro. In vivo experiments further validated the oncogenic functions of *LNBC3*, as xenograft tumour growth and proliferation were enhanced with increasing *LNBC3* levels [83]. Mechanistic studies revealed that *LNBC3* interacted with BCL6, leading to the stabilization of BCL6 by reducing proteasomal degradation [83]. These findings suggest that the *LNBC3*–BCL6 axis may serve as a potential target for pharmaceutical intervention in NSCLC.

Furthermore, higher *LSINCT5* levels were associated with malignant clinicopathological features and poor survival outcomes [84]. Functional assays demonstrated that *LSINCT5* promoted migration and viability of NSCLC cells in vitro. In vivo experiments further confirmed the role of *LSINCT5* in promoting NSCLC progression. Mechanistically, *LSINCT5* was found to interact with HMGA2, stabilizing this oncogenic factor by inhibiting proteasome‐mediated degradation. These findings suggest that *LSINCT5* may contribute to NSCLC tumourigenesis through the *LSINCT5*–HMGA2 axis. Targeting this axis could potentially be explored as a therapeutic approach for NSCLC [84].

Recent studies have identified the lncRNA *LINC01833* as a significant contributor to NSCLC progression [85]. Elevated expression of *LINC01833* has been observed in NSCLC tissues, and its overexpression has been shown to promote proliferation, migration, and invasion of NSCLC cells [86].

Mechanistic investigations have revealed that *LINC01833* exerts its effects by modulating the N6‐methyladenosine (*m6A*) modification of target genes. RNA pulldown and *m6A*‐specific immunoprecipitation assays have provided evidence of the interaction between *LINC01833* and methyltransferase 3 (METTL3), a key enzyme involved in *m6A* modification [85]. Moreover, *LINC01833* has been found to form a complex with heterogeneous nuclear ribonucleoprotein A2/B1 (HNRNPA2B1) in NSCLC, further highlighting its involvement in cancer progression [85].

Moreover, the lncRNA *MALAT1* has been shown to regulate proliferation, apoptosis, migration and invasion in NSCLC [87].

### 5.20 The Role of lncRNAs in Chronic Kidney Disease (CKD)

CKD is a progressive condition characterized by the gradual loss of kidney function over time. It is often associated with various risk factors, including hypertension, diabetes and obesity [88]. CKD can lead to serious complications, such as end‐stage renal disease (ESRD), cardiovascular disease and metabolic disorders [88, 89].

Recent studies have highlighted the regulatory roles of lncRNAs in CKD pathogenesis [90]. In a study it was demonstrated that a novel lncRNA, *CYP4B1‐PS1‐001*, was significantly downregulated in early diabetic nephropathy [91]. This downregulation was observed both in vivo and in vitro. Further investigation revealed that *CYP4B1‐PS1‐001* inhibited the proliferation and fibrosis of mouse mesangial cells (MMCs) [91]. The study also identified an interaction between *CYP4B1‐PS1‐001* and the upregulated protein nucleolin (NCL). Moreover, the degradation of *CYP4B1‐PS1‐001‐*associated NCL was mediated by a ubiquitin proteasome‐dependent pathway [91].

Identification of reliable prognostic biomarkers is crucial for predicting disease progression and guiding therapeutic interventions in CKD. Several studies have investigated the potential of lncRNAs as prognostic markers in CKD [90, 92].

In another study, the expression of the lncRNA *MIR4435‐1HG* was found to be upregulated in renal cell carcinoma [68]. This upregulation was correlated with TNM stage, tumour size and Fuhrman grade. High expression of *MIR4435‐1HG* was also associated with poor prognosis. Functional experiments revealed that *MIR4435‐1HG* knockdown inhibited cell proliferation, migration and invasion of renal carcinoma cells. Further analysis using RNA pull‐down and western blotting identified an interaction between *MIR4435‐1HG* and pyruvate carboxylase [68].

To understand the mechanisms underlying the roles of lncRNAs in CKD, researchers have employed various experimental techniques. RNA–protein pulldown assays, RNA‐binding protein immunoprecipitation and MS have been utilized to study the interactions between lncRNAs and proteins involved in CKD pathogenesis [93, 94, 95].

### 5.21 lncRNAs in Diabetic Kidney Disease (DKD)

DKD is a common complication of diabetes and a leading cause of ESRD. miRNAs and lncRNAs have been implicated in the pathogenesis of DKD [96]. A study by Kato et al. focused on the *miR‐379* megacluster of miRNAs and its host transcript lnc‐megacluster (*lncMGC*) in DKD [96]. They identified *lncMGC*‐interacting proteins through RNA pulldown experiments and MS. The study also created lncMGC‐knockout mice and examined the effects of *lncMGC* on gene expression, histone modifications and chromatin remodelling in primary MMCs [96]. The results indicated that *lncMGC* interacts with nucleosome remodelling factors to promote chromatin relaxation and enhance the expression of *lncMGC* itself and other genes involved in DKD.

## 6 Conclusion

As technology continues to evolve, MS is expected to further advance in its capabilities and applications. The expansion of databases containing spectra of known organisms will enhance the accuracy and reliability of species identification. Additionally, ongoing research aims to refine the technique's ability to detect and analyse specific biomarkers associated with various diseases and conditions. These advancements will contribute to the development of personalized medicine and precision diagnostics, paving the way for improved patient care. Therefore, MALDI‐TOF and other nominated MS methods are versatile and powerful analytical techniques that have revolutionized the identification and analysis of biomolecules. With its applications in proteomics, metabolomics, lipidomics, genomics and glycomics, this technology provides valuable insights into various omics fields. Despite its limitations, the continuous advancements in technology and databases are expected to further enhance the capabilities and applications of MALDI‐TOF MS, shaping the future of biomedical research and clinical care.

MS can be used to study the properties of lncRNAs, including their tertiary structure, interactions with other molecules and PTMs such as phosphorylation and methylation. It can also be used to study the interactions of lncRNAs with other molecules, such as proteins, DNA and RNA. In addition to studying the properties of lncRNAs, MS can also be used to identify lncRNAs in complex biological samples, such as cells and tissues. This can be done by first extracting the RNA from the sample and then using MS to identify and quantify the lncRNAs present.

## Author Contributions

All authors contributed to the study conception, and this review was designed by Mina Moradi and Mohammad Mehdi Akbarin. Data collection and analysis were performed by Mina Moradi, Zahra Farjami and Mohammad Mehdi Akbarin. The first draft of the manuscript was written by Mina Moradi and Mohammad Mehdi Akbarin, and all authors commented on previous versions of the manuscript. The first draft was edited by Mohammad Mehdi Akbarin. All authors read and approved the final manuscript.

## Ethics Statement

The authors have nothing to report.

## Consent

All authors have read and understand the provided information about their role in participation in this study. The authors declared that all mentioned information, tables and images are legally extracted from the open access source. The authors officially state their consent for the publication of all details, including the text and figures which are mentioned in this article.

## Conflicts of Interest

The authors declare no conflicts of interest.

## Data Availability

The data that support the findings of this study are available from the corresponding author upon reasonable request.
